# Extracorporeal cardiopulmonary resuscitation for patients with refractory out-of-hospital cardiac arrest: a propensity score matching, observational study

**DOI:** 10.1038/s41598-024-60620-7

**Published:** 2024-04-30

**Authors:** Hong-Mo Shih, Wei-Jun Lin, You-Cian Lin, Shih-Sheng Chang, Kuan-Cheng Chang, Shao-Hua Yu

**Affiliations:** 1 Department of Emergency Medicine, China Medical University Hospital, 2 Yue-Der Road, Taichung City, Taiwan; 2 Division of Cardiovascular Medicine, Department of Internal Medicine, China Medical University Hospital, Taichung, Taiwan; 3 Surgical Department Cardiovascular Division, China Medical University Hospital, Taichung, Taiwan; 4 School of Medicine, College of Medicine, China Medical University, Taichung, Taiwan

**Keywords:** Cardiology, Cardiovascular diseases, Prognosis

## Abstract

Extracorporeal cardiopulmonary resuscitation (ECPR) is increasingly performed as an adjunct to conventional cardiopulmonary resuscitation (CCPR) for refractory out-of-hospital cardiac arrest (OHCA). However, the specific benefits of ECPR concerning survival with favorable neurological outcomes remain uncertain. This study aimed to investigate the potential advantages of ECPR in the management of refractory OHCA. We conducted a retrospective cohort study involved OHCA patients between January 2016 and May 2021. Patients were categorized into ECPR or CCPR groups. The primary endpoint assessed was survival with favorable neurological outcomes, and the secondary outcome was survival rate. Multivariate logistic regression analyses, with and without 1:2 propensity score matching, were employed to assess ECPR’s effect. In total, 1193 patients were included: 85underwent ECPR, and 1108 received CCPR. Compared to the CCPR group, the ECPR group exhibited notably higher survival rate (29.4% vs. 2.4%; *p* < 0.001). The ECPR group also exhibited a higher proportion of survival with favorable neurological outcome than CCPR group (17.6% vs. 0.7%; *p* < 0.001). Multivariate logistic regression analysis demonstrated that ECPR correlated with increased odds of survival with favorable neurological outcome (adjusted odds ratio: 13.57; 95% confidence interval (CI) 4.60–40.06). Following propensity score matching, the ECPR group showed significantly elevated odds of survival with favorable neurological outcomes (adjusted odds ratio: 13.31; 95% CI 1.61–109.9). This study demonstrated that in comparison to CCPR, ECPR may provide survival benefit and increase the odds of favorable neurological outcomes in selected OHCA patients.

## Introduction

Out-of-hospital cardiac arrest (OHCA) is the leading cause of death and disability worldwide^1^. Despite advancements in the chain of survival and improvements in cardiopulmonary resuscitation (CPR), the post-OHCA prognosis remains unsatisfactory. For patients receiving conventional CPR (CCPR), statistics reveal a return of spontaneous circulation (ROSC) at 29.7%, survival to hospital admission at 22.0%, and survival to hospital discharge at 8.8%^2^. In Taiwan, post-resuscitation, only 24.9% exhibited ROSC, 7.8% survived to discharge, with only 3.7% achieve a favorable neurological outcome, with a mere 1% of CPR recipients experiencing a Cerebral Performance Category (CPC) score of 1 or 2 after enduring CPR for more than 35 minutes^3^.

Extracorporeal CPR (ECPR) through oxygenation and pumping units was initially proposed in 1976 for cardiac arrest patients^4^. It has increasingly been adopted as an adjunct to CCPR for refractory cardiac arrest^5^. Observational studies highlight survival benefits associated with ECPR^6,7^, particularly among OHCA patients with short low-flow duration, shockable rhythms, higher arterial pH value, and lower serum lactate levels^8^. However, a 2020 Paris registry study reported no discernible difference in post-OHCA survival rates between ECPR and CCPR^9^. The 2020 ARREST trial, the first randomized controlled trial assessing ECPR, demonstrated that early extracorporeal membrane oxygenation (ECMO)–facilitated resuscitation for OHCA patients with refractory ventricular fibrillation significantly improved survival to hospital discharge compared to standard advanced life support treatment^10^. Recent meta-analyses have also supported ECPR’s potential to enhance survival and long-term favorable neurologic outcomes in OHCA patients^11,12^. Nevertheless, subsequent randomized controlled trials reported no significant disparities between CCPR and ECPR in terms of survival with favorable neurological outcomes^13,14^.

These varying findings suggest that while a subset of patients with refractory arrest benefit from ECPR, unnecessary ECPR engagement may lead to avoidable complications and heightened medical costs. Given the substantial uncertainty in relevant evidence, the 2021 European Resuscitation Guidelines only weakly recommend ECPR in the case of unsuccessful CCPR^15^. Consequently, this present study aims to investigate the potential advantages of ECPR in achieving favorable outcomes among patients who experienced refractory OHCA.

## Methods

### Study design and setting

This retrospective cohort study involved individuals admitted to China Medical University Hospital (CMUH) in Taichung, Taiwan, subsequent to experiencing OHCA. CMUH serves as an urban tertiary medical center, witnessing an annual influx of 140,000–160,000 emergency department (ED) visits, with over 400 cases involving OHCA patients receiving CPR. In Taiwan, emergency medical service (EMS) personnel provide prehospital resuscitation, encompassing chest compressions, airway management using bag–valve–mask ventilation or laryngeal mask airway, and defibrillation with automated external defibrillators^16^. Upon arrival at CMUH’s ED, OHCA patients receive advanced life support adhering to international guidelines^17,18^, inclusive of endotracheal intubation, CPR, epinephrine administration, and electrical defibrillation. Patients suspected of cardiac causes triggering cardiac arrest, such as shockable rhythms during CPR or ST-elevation myocardial infarction post-ROSC on electrocardiogram, undergo percutaneous coronary intervention. Target temperature management is contemplated for patients achieving ROSC exhibiting impaired consciousness (Glasgow Coma Scale score: < 9) or inability to follow commands.

### Ethical declarations

This retrospective study was reviewed and approved by the Research Ethics Committee, China Medical University & Hospital, Taichung, Taiwan (CMUH REC No.: CMUH109-REC2-182). The need for informed consent from study participants was waived by the Research Ethics Committee due to the retrospective nature of the study and the use of de-identified data, ensuring participant anonymity and confidentiality. All procedures were executed in accordance with ethical standards outlined in the Helsinki Declaration of 1975.

### Patient selection

Patients included in the study were those experiencing cardiac arrest and receiving resuscitation at CMUH’s ED between January 2016 and May 2021. Exclusions comprised individuals aged < 20 years, cases of cardiac arrest attributable to circumstantial causes such as trauma, hanging, drowning, intoxication or asphyxia, transferred from other hospitals, and patients without CPR attempted at the ED. Additionally, patients who achieved sustained ROSC within 15 min of advanced life support at the ED were also excluded from this study.

### ECPR and relevant variables

OHCA patients were categorized into ECPR and CCPR groups based on whether they received adjunct ECPR. ECPR initiation was activated by an emergency physician and performed by cardiovascular surgeons after verification. Although definitive criteria were absent, consideration for ECPR typically involved patients aged < 65 years, suspected to have arrest from cardiac causes (e.g., acute myocardial infarction and pulmonary embolism), receipt of bystander CPR after collapsing, presentation with shockable rhythms, and an estimated collapse-to-ECMO time of < 100 min.

The Taichung Sudden Cardiac Arrest Registry prospectively registers all OHCA patients following an Utstein-style template^19–21^. The database encompassed demographic details (age and sex; personal medical history), prehospital variables (witness of collapse, location, bystander CPR, and time record of prehospital EMS resuscitation), initial rhythm (shockable or non-shockable rhythm), in-hospital resuscitation parameters (arrival time, initial rhythm, CPR duration, ROSC time), and post-resuscitation care data (target temperature management, percutaneous coronary intervention).

### Study outcomes and statistical analysis

The primary outcome assessed was survival with a favorable neurological outcome, defined as a CPC score of 1 or 2 (CPC of 3–5 was defined as unfavorable neurological outcome)^22^. The secondary outcome was survival rate, defined as survival to hospital discharge (including those transferred to a rehabilitation facility or extended care facility and those requiring home nursing services) or survival for > 30 days^23^..

Statistical analyses were performed using SAS (version 9.4; SAS Institute, Cary, NC, USA). Significance was set at *p* value of < 0.05. Categorical variables are presented in terms of numbers and percentages and were compared using the chi-square test. Continuous variables are presented in terms of the median and interquartile range values and were analyzed with Mann–Whitney U test. For the primary outcome, bivariate analysis with the chi-square and Mann–Whitney U tests were performed to identify factors associated with favorable outcome. Factors present before the decision to activate ECPR and those that might influence post-OHCA prognosis were selected for multivariate analysis. A multivariate logistic regression model adjusted for age, OHCA location, bystander CPR, witness of collapse, and initial shockable rhythm was used to evaluate the effect of ECPR on outcomes after OHCA. Propensity score matching was performed for the ECPR and CCPR groups (at a 1:2 ratio) by adjusting for the factors present before the decision of ECPR activation; these factors included age, sex, location, bystander CPR, witness, and shockable rhythms. Propensity score matching was performed using the nearest neighbor method, with the caliper width set at 0.2. After matching, the standardized mean difference was calculated to assess the balance between the ECPR and CCPR groups regarding the covariates. Additionally, the C-statistic was calculated to evaluate the performance of the propensity score matching model. Conditional logistic regression without further adjustment was performed to investigate the effect of ECPR on favorable neurological outcomes after OHCA. Patients were further stratified based on potential prognostic factors following OHCA. Subgroup analyses were conducted using conditional logistic regression for the matched cohort and depicted in a forest plot.

## Results

**Figure 1:**
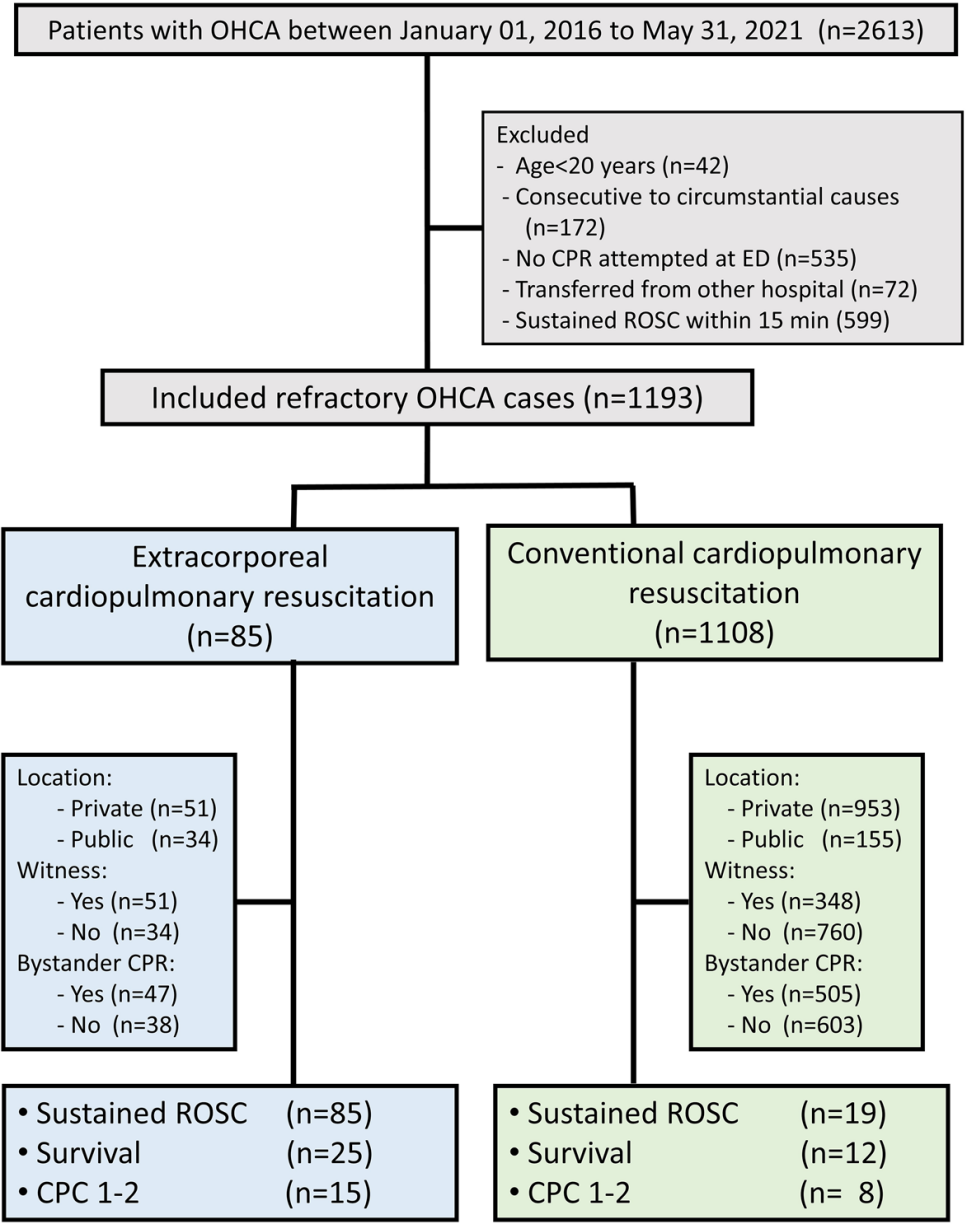
Flowchart for patient enrollment. OHCA, out-of-hospital cardiac arrest; ROSC, return of spontaneous circulation; CPR: cardiopulmonary resuscitation; ED: emergency department; CPC, cerebral performance category.

From January 2016 to May 2021, a total of 2613 OHCA patients were admitted to CMUH’s ED. Of these, 1420patients were excluded due to age < 18 years (n = 42), cardiac arrest due to circumstantial causes (n = 172), no CPR attempted at ED (n = 535), transferred from another hospital (n = 72) or achieving sustained ROSC within 15 min at ED (n = 599). The final cohort comprised 1193 patients, with 85 undergoing ECPR and 1108 receiving CCPR. Among them, 104 patients (8.72%) achieved sustained ROSC, and 23 (1.93%) had a favorable neurological outcome (Fig. 1).

### Baseline characteristics

Table 1 displays patients’ baseline characteristics. The ECPR group exhibited a younger age (56.0 vs. 71.0 years, *p* < 0.001), male sex (83.5% vs. 62.5%, *p* < 0.001) and a higher frequency of OHCA occurrence in public locations (40.0% vs. 14.0%, *p* < 0.001). Additionally, a higher proportion of patients in the ECPR group experienced witnessed arrests and initial shockable rhythms. Compared to the CCPR group, the ECPR cohort displayed significantly higher rates of sustained ROSC (100.0% vs. 10.4%, *p* < 0.001), higher proportion of survival (29.4% vs. 2.4%, *p* < 0.001) and survival with a favorable neurological outcome (17.6% vs. 0.7%, *p* < 0.001).

**Table 1:** Characteristics of the ECPR and CCPR groups, before and after propensity score matching.

Variables	Original cohort			1:2 propensity score matching		
	ECPR (n = 85)	CCPR (n = 1108)	*p *value	ECPR (n = 77)	CCPR (n = 154)	SMD
Age	56.0 (45.0–64.0)	71.0 (58.0–82.0)	< 0.001	57.0 (47.0–65.0)	56.0 (44.0–66.0)	0.027
Male sex	71 (83.5)	693 (62.5)	< 0.001	63 (81.8)	124 (80.5)	0.033
Prehospital time, min	23.0 (20.0–26.0)	22.0 (19.0–26.0)	0.784	23.0 (20.0–26.0)	22.0 (19.0–26.0)	0.018
Public location arrest	34 (40.0)	155 (14.0)	< 0.001	30 (39.0)	52 (33.8)	0.108
Bystander CPR	47 (55.3)	505(45.6)	0.083	44 (57.1)	73 (47.4)	0.196
Witness	51 (60.0)	348(31.4)	< 0.001	47 (61.0)	89 (57.8)	0.066
Initial shockable rhythm	74 87.1)	388(35.0)	< 0.001	66 (85.7)	135 (87.7)	0.057
Comorbidities						
Diabetes mellitus	23 (27.1)	276 (24.9)	0.659	21 (27.3)	27 (17.5)	0.235
Hypertension	25 (29.4)	354 (32.0)	0.628	24 (31.2)	33 (21.4)	0.223
Cerebrovascular disease	4 (4.7)	77 (7.0)	0.428	4(5.2)	9(5.8)	0.028
Coronary artery disease	14 (16.5)	134 (12.1)	0.238	13 (16.9)	18 (11.7)	0.149
Congested heart failure	2 (2.4)	67 (6.1)	0.225	2 (2.6)	8 (5.2)	0.135
Liver cirrhosis	0 (0.0)	13 (1.2)	0.616	0 (0.0)	3 (1.9)	0.199
End-stage renal disease	6 (7.1)	78 (7.0)	0.994	6 (7.8)	8 (5.2)	0.106
Cancer	1 (1.2)	120 (10.8)	0.004	1 (1.3)	13 (8.4)	0.337
CPR duration, min, Median, IQR	47.0 (36.0–60.0)	31.0 (29.0–38.0)	< 0.001	46.0 (34.0–60.0)	32.0 (30.0–44.0)	0.133
Collapse to ECMO flow, min, Median, IQR	92.0 (76.0–118. 0)			93.0 (76.0–114.0)		
Sustained ROSC	85 (100.0)	115 (10.4)	< 0.001	77 (100.0)	19 (12.3)	3.770
Survival discharge	25 (29.4)	26 (2.4)	< 0.001	22 (28.6)	12 (7.8)	0.559
Neurological outcome			< 0.001			0.413
CPC 1–2	15 (17.6)	8 (0.7)		14 (18.2)	8 (5.2)	
CPC 3–5	70 (82.4)	1100 (99.3)		63 (81.8)	146 (94.8)	
Post ROSC management						
Coronary angiography	73 (85.9)	25 (2.3)	< 0.001	66 (85.7)	10 (6.5)	2.618
TTM	55 (64.7)	33 (3.0)	< 0.001	51 (66.2)	14 (9.1)	1.460

CPR: cardiopulmonary resuscitation; IQR: interquartile range; ECMO: extracorporeal cardiopulmonary membrane oxygenation; ROSC: return of spontaneous circulation; CPC: cerebral performance category, TTM: targeted temperature management.

### Factors associated with favorable outcome

Table 2 presents bivariate analysis for factors potentially associated with favorable neurological outcomes. Patients with refractory OHCA who survived with favorable neurological function were younger, had a higher proportion of witnessed arrests, and presented with initial shockable rhythms. Patients who received ECMO and TTM also had a higher likelihood of achieving favorable outcomes.

**Table 2:** Factors associated with a favorable outcome among patients with refractory out-of-hospital cardiac arrest.

Variables	Neurological outcome		*p*-value
	Favorable outcome (n = 23)	Unfavorable outcome (n = 1170)	
Age	53.0 (37.0–57.0)	70.0 (57.0–82.0)	< 0.001
Male sex	17 (73.9)	747 (63.8)	0.319
Prehospital time, min	21.0 (20.0–23.0)	22.0 (19.0–26.0)	0.258
Public location arrest	7 (30.4)	182 (15.6)	0.076
Bystander CPR	14 (60.9)	538 (46.0)	0.156
Witness	13 (56.5)	386 (33.0)	0.017
Initial shockable rhythm	17 (73.9)	445 (38.0)	< 0.001
Comorbidities			
Diabetes mellitus	6 (26.1)	293 (25.0)	0.908
Hypertension	5 (21.7)	374 (32.0)	0.296
Cerebrovascular accident	1 (4.4)	80 (6.8)	1.000
Coronary artery disease	4 (17.4)	144 (12.3)	0.516
Congested heart failure	1 (4.4)	68 (5.8)	1.000
Liver cirrhosis	1 (4.4)	12 (1.0)	0.224
End-stage renal disease	2 (8.7)	82 (7.0)	0.673
Cancer	0 (0.0)	121 (10.3)	0.158
CPR duration, min, median (IQR)	37.0 (24.0–51.0)	31.0 (30.0–40.0)	0.479
Coronary angiography	19 (82.6)	79 (6.8)	< 0.001
TTM	18 (78.3)	70 (6.0)	< 0.001
ECMO	15 (65.2)	70 (6.0)	< 0.001

CPR: cardiopulmonary resuscitation; IQR: interquartile range; ECMO: extracorporeal cardiopulmonary membrane oxygenation; TTM: targeted temperature management.

### Propensity score matching and conditional logistic regression

Following 1:2 propensity score matching, the ECPR and CCPR groups included 77 and 154 patients, respectively, with a C-statics of 0.88. After matching, the ECPR group exhibited higher rates of survival (28.6% vs. 7.8%), and survival with a favorable neurological outcome (18.2% vs. 5.2%) compared to the CCPR group (Table 1).

### Multivariate analysis for outcome after refractory OHCA

**Figure 2:**
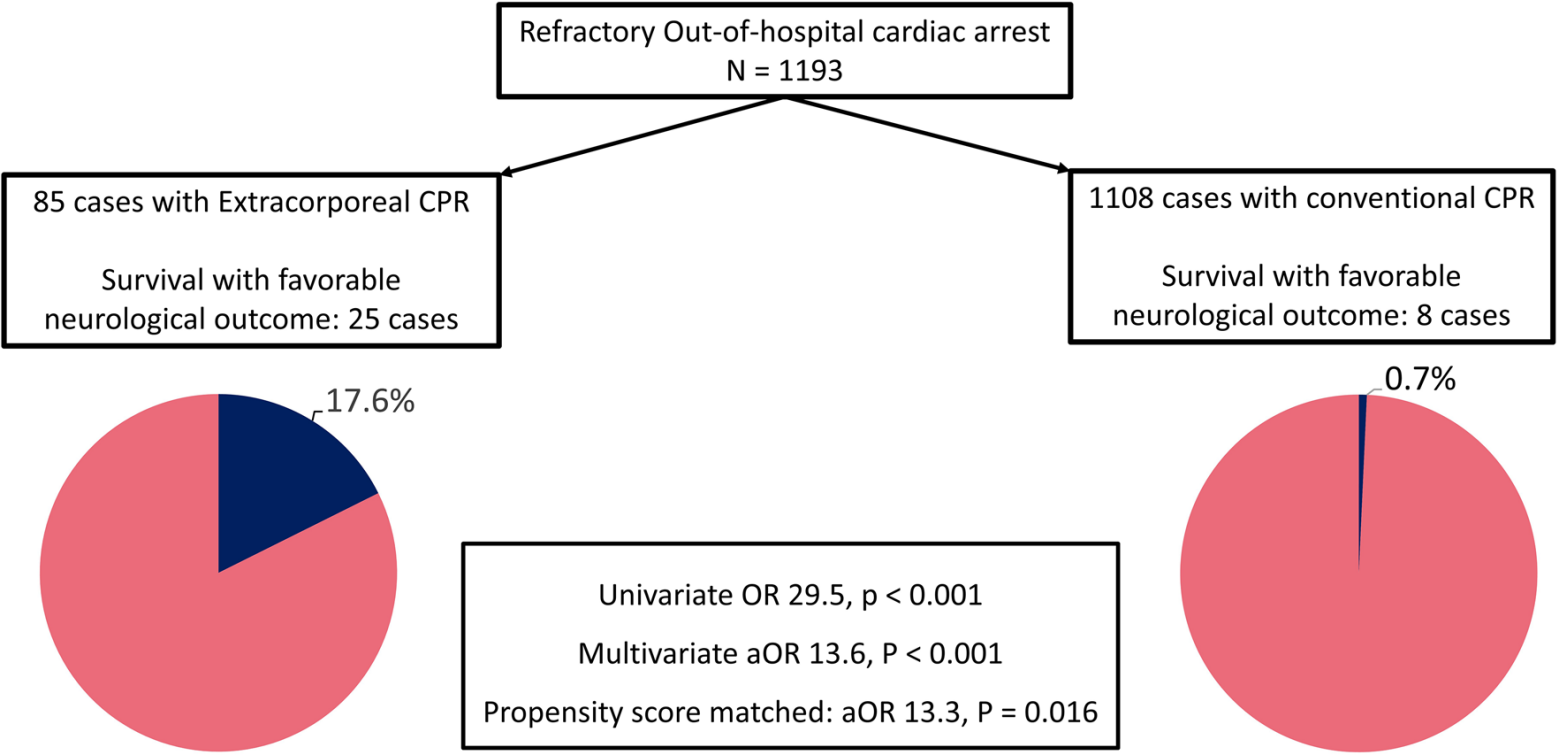
Survival with a favorable neurological outcome among patients with refractory out-of-hospital cardiac arrest receiving extracorporeal or conventional CPR. Among 1193 patients with out-of-hospital cardiac arrest, 85 received extracorporeal CPR. These patients demonstrated a higher proportion of survival with a favorable neurological outcome than those receiving conventional CPR. CPR, cardiopulmonary resuscitation.

Multivariate logistic regression for prognosis after refractory OHCA is presented in Table 3. In the original cohort, the ECPR group demonstrated a better chance of survival (adjusted odds ratio [aOR]: 7.84; 95% confidence interval [CI]: 3.83–16.04) as well as favorable neurological outcomes (aOR: 13.57; 95% CI: 4.60–40.06). Following propensity score matching, the ECPR group continued to exhibit a higher proportion of favorable neurological outcomes (aOR: 13.31; 95% CI: 1.61–109.9) and survival rates (aOR: 6.02; 95% CI: 2.19–16.52) in the conditional logistic regression analysis. Figure 2 summarized the comparison of primary outcome between the ECPR and CCPR groups.

**Table 3:** Multivariate analysis for patient outcomes before and after propensity score matching.

	Original cohort			Matched cohort		
	CCPR	ECPR	aOR (95% CI)	CCPR	ECPR	aOR (95% CI)
	n/N (%)	n/N (%)		n/N (%)	n/N (%)	
Primary outcome						
Favorable neurological outcome	8/1108 (0.7)	15/85 (17.6)	13.57 (4.60–40.06)	8/154 (5.2)	14/77 (18.2)	13.31 (1.61–109.9)
Secondary outcome						
Survival	26/1108 (2.3)	25/85 (29.4)	7.84 (3.83–16.04)	12/154 (7.8)	22/77 (28.6)	6.02 (2.19–16.52)

ECPR, extracorporeal cardiopulmonary resuscitation; CCPR, conventional cardiopulmonary resuscitation.

### Factors affecting neurological outcome in ECPR patients

Table 4 illustrates factors influencing a favorable neurological outcome in ECPR patients. Younger age (48.0 vs. 58.5 years, *p* = 0.001), CPR duration (37.0 vs. 51.0 min, p = 0.006) and collapse to ECMO flow initiation time (76.0vs. 98.0 min, *p* = 0.031) were associated with higher rates of a favorable neurological outcome.

**Table 4:** Factors associated with a favorable outcome in refractory out-of-hospital cardiac arrest patients receiving extracorporeal cardiopulmonary resuscitation.

Variables	Favorable outcome (n = 15)	Unfavorable outcome (n = 70)	*p*-value
Age	48.0 (34.0–57.0)	58.5 (46.0–66.0)	0.005
Male sex	13 (86.7)	58 (82.9)	1.000
Prehospital time, min	21.0 (20.0–23.0)	23.0 (20.0–26.0)	0.119
Public location arrest	4 (26.7)	30 (42.9)	0.245
Bystander CPR, yes	8 (53.3)	39 (55.7)	0.866
Witness, yes	7 (46.7)	44 (62.9)	0.245
Initial shockable rhythm	10 (66.7)	64 (91.4)	0.021
Any ROSC before ECMO	7 (46.7)	22 (31.4)	0.258
CPR duration, minutes	37.0 (28.0–43.0)	51.0 (38.0–70.0)	0.006
Collapse to ECMO team activation, min	29.0 (27.0–36.0)	33.5 (29.0–43.5)	0.064
ECMO team activation to flow initiation, min	55.0 (35.0–79.0)	56.0 (44.0–74.0)	0.648
Collapse to ECMO flow initiation, min	76.0 (63.0–98.0)	98.0 (79.0–118.0)	0.031
Blood pH before ECMO	7.1 (6.9–7.2)	7.0 (6.9–7.1)	0.556
Post resuscitation management			
Coronary angiography	15(100.0)	58 (82.9)	0.113
TTM	12(80.0)	43 (61.4)	0.172

CPR: cardiopulmonary resuscitation; ROSC: return of spontaneous circulation; ECMO: extracorporeal cardiopulmonary membrane oxygenation; TTM: targeted temperature management.

### Subgroup analysis

**Figure 3:**
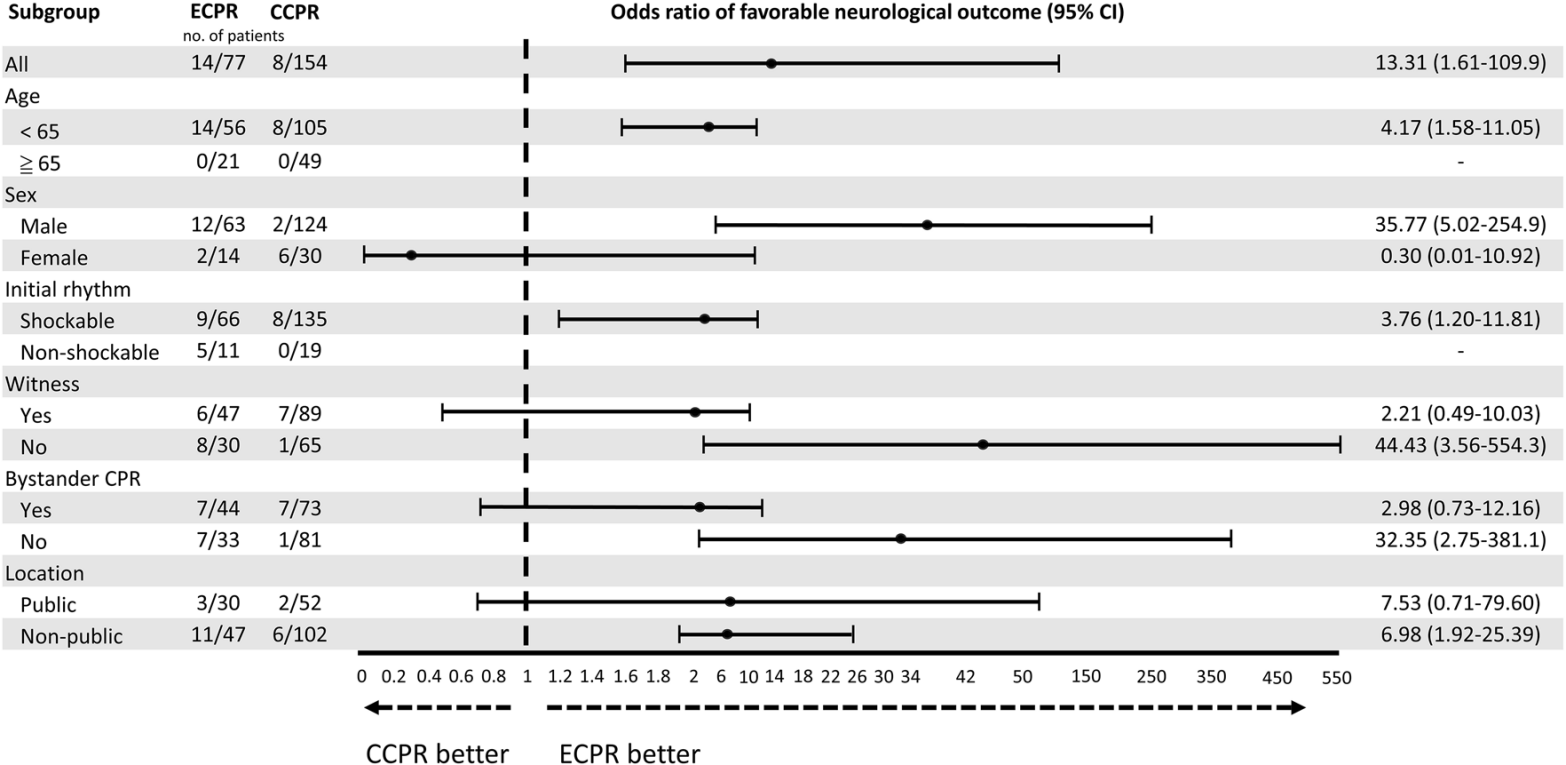
Forrest plot of subgroup analysis for favorable neurological outcome. Shown are the analysis of primary outcome (survival with a favorable neurological function) in prespecified subgroups. The forest plot shows the adjusted odds ratio derived from the multivariate logistic regression. The horizonal bar represent 95% confidence intervals. ECPR, extracorporeal cardiopulmonary resuscitation; CCPR, conventional cardiopulmonary resuscitation. ECPR, extracorporeal cardiopulmonary resuscitation; CCPR, conventional cardiopulmonary resuscitation.

The effect of ECPR on favorable neurological outcomes after propensity score matching was illustrated in Fig. 3. A greater likelihood of achieving a favorable outcome was observed in the ECPR group among patients aged < 65 years, males, and those with an initial shockable rhythm. However, there was no significant difference in prognosis between ECPR and CCPR for patients with witnessed arrest, those who received bystander CPR, or those who experienced arrest in public places. Conversely, the ECPR group demonstrated improved outcomes among patients without a witnessed arrest, those who did not receive bystander CPR, and those whose arrest occurred in non-public locations.

## Discussion

Our investigation revealed higher survival rate and a greater probability of survival with a favorable neurological outcome in refractory OHCA patients who underwent ECPR compared to those receiving CCPR. This study represents a relatively large single-center inquiry into the efficacy of ECPR in treating refractory OHCA patients and is among the few to use propensity score matching. Even after propensity score matching, the distinction in prognosis between the CCPR and ECPR groups persisted significantly in the multivariate analysis.

Evidence suggests that patients with refractory OHCA can benefit from ECPR. Retrospective studies utilizing propensity score matching suggested that OHCA patients undergoing ECPR have more favorable prognosis than those who are treated with CCPR^7,24,25^. A recent meta-analysis of randomized and propensity score matching studies revealed that 14% of all patients in the ECPR group survived with a favorable neurological outcome compared with only 7.8% in the CCPR group^12^. Consistent with these findings, our results from this single-center observational study, utilizing propensity score matching, indicated that ECPR can improve post-OHCA prognosis. Despite the limitations of a single-center study with a relative smaller sample size, it advantages lie in standard care protocols, complete patient data, consistent medical care quality, precise timing, and definitive diagnosis. These factors were considered in our propensity score matching, which facilitated an objective comparison of the ECPR and CCPR groups in terms of outcomes.

The ARREST trial reported a higher survival to hospital discharge rate in ECPR group compared to the CCPR group^10^. However, subsequent randomized controlled trials such as the Prague trial^26^ and the INCEPTION trial^14^ indicated no discernible differences in neurological recovery between the two groups. This discrepancy may be due to the differences in the time from collapse to ECMO flow, which was 59 min in the ARREST trial but 62 and 74 min in the Prague and INCEPTION trials, respectively. In our study, this duration was notably longer at 92 min, much longer than these trials as well as that reported in a Korean nationwide registry study^27^. This was primarily because of the longer time from ECMO team activation to the start of ECMO flow in our study than in the other studies; this delay is caused mainly by our protocol of surgeon verification for ECMO implantation, which involves discussion with the patient’s family, rather than direct activation. Although our findings revealed substantial benefits of ECPR, survival rate and favorable neurological outcomes were lower in our study than in the ARREST and Prague trials. Thus, a prepared ECMO team and protocolized team activation process may be beneficial.

Divergence among previous studies could also arise from discrepancies in the inclusion criteria for ECPR activation. For instance, the ARREST trial included only patients with ventricular arrhythmia failing to achieve ROSC after three defibrillation attempts^10^. The INCEPTION trial also included patients with ventricular arrhythmia but enrolled those who experienced refractory cardiac arrest despite 15 min of advanced life support^14^. On the other hand, the Prague trial enrolled both patients with ventricular arrhythmia and those without it, defining refractory arrest as failure to achieve ROSC after 5 min of advanced life support^26^. In current study, we included patients without sustained ROSC after 15 min of advanced life support and demonstrated the benefit of ECPR on favorable after OHCA, especially in patients younger than 65 years old, male sex and presenting with initial shockable rhythms. However, among patients with witnessed arrest, bystander CPR, and arrest at a public location, the proportions of surviving with favorable neurological outcome were comparable between the CCPR and ECPR groups This could be due to the influence of various factors on patient outcomes, such as the quality of chest compressions, duration of no-flow and low-flow time, early defibrillation, and the cause of cardiac arrest.^28–30^. Additionally, the ECPR activation was at the discretion of physicians, prioritizing patients with a relatively high likelihood of achieving a favorable outcome. This decision-making process may also confound the effect of ECPR on neurological outcomes. Collectively, findings from the Prague trial, INCEPTION trial, Paris registry study, and our investigation suggest that activation of ECPR may provide benefit for selected patient of refractory OHCA.

We also identified factors predictive of neurological recovery in ECPR patients. Consistent with previous study^31^, the median CPR duration in our study was significantly shorter among patients with a favorable neurological outcome (37.0 min) than among those with an unfavorable neurological outcome (51.0 min). Furthermore, we observed younger age, and shorter collapse to ECMO flow initiation, were associated with favorable neurological outcomes, consistent with the results of previous study^32^. Age emerged as a crucial prognostic factor across all OHCA patients^19^. We observed that in the ECPR group, patients exhibiting neurological recovery tended to be younger than those not exhibiting neurological recovery.

Nonetheless, our study presents several limitations. Being retrospective, the study lacked stringent criteria for ECPR activation, leading to varied demographic characteristics, comorbidities, and prognostic parameters between the ECPR and CCPR groups. The decision-making process regarding whether to activate ECPR, the presence of signs of life, and the level of end-tidal carbon dioxide were not documented in the electronic medical records. Despite propensity score matching, potential selection bias cannot be completely rule out. Moreover, due to the limited ECPR cases, statistical analysis might have lacked sufficient power to discern differences in prognostic factors within the ECPR group. The longer time from collapse to ECMO flow in our study compared to previous studies might also impact the outcomes. At our institute, the consensus time window for ECPR is < 100 min from collapse to ECMO flow. A nationwide multicenter study from Denmark, which also used the consensus criteria for ECPR with a similar median low-flow time of 105 min, reported a high survival rate with favorable neurological outcome^34^; this finding is consistent with ours. The present study may serve as a valuable reference for institutes without strict activation criteria and for patients with OHCA requiring transportation for ECMO. Lastly, despite the advantage of consistency in resuscitation and postresuscitation care, the use of a single-center registry limits the generalizability of our results. Future larger-scale multicenter studies or those using nationwide databases are warranted.

## Conclusions

Base on the current propensity score–matched analysis, ECPR may enhance the odds of survival with favorable neurological outcome in selected patients with refractory OHCA. Additional investigation is required to determine optimal criteria for identifying appropriate candidates for ECPR.

## Data Availability

The data that support the findings of this study are available from the corresponding author upon reasonable request.
